# Comparative Study of Hypoglycemic Activity of Cr-Gliclazide and Its β‑Cyclodextrin Inclusion Complex

**DOI:** 10.1021/acsomega.6c09131

**Published:** 2026-10-03

**Authors:** Khalid Rasheed, Christy Munir, Anum Hayat, Amna Yousaf, Faryal Chaudhry, Meerab Fatima

**Affiliations:** † Department of Chemistry, Quaid-e-Azam University, Islamabad 15320, Pakistan; ‡ Department of Chemistry, Forman Christian College (A Chartered University), Zahoor Elahi Road, Gulberg III, Lahore 54600, Pakistan; § Department of Chemistry, 119706 Kinnaird College for Women, 93 Jail Road, Lahore 54000, Pakistan

## Abstract

This study investigated the synthesis and pharmacological properties of chromium metal complexes with sulfonylureas (hypoglycemic agents). In these complexes, the structural element (chromium) is held within the cavity of the β-cyclodextrin (βCD) molecule. Conditions were optimized for the inclusion complex: 1:1 host–guest ratio, pH = 12, and temperature: ∼25 °C. Cr­(III)-Gliclazide (GCZ) and βCD under appropriate experimental conditions show circular dichroism (CD) bands in the visible region: positive Cotton bands at 24,691 cm^–1^ (Δε = 20.103 degree L mol^–1^ cm^–1^) and 16,666 cm^–1^ (Δε = 26.6 × 10^–3^ degree L mol^–1^ cm^–1^) and a strong negative Cotton band at 19,230 cm^–1^ (*A*ε = 38.1 × 10^–3^ degree L mol^–1^ cm^–1^). The synthesized complexes were assessed for their hypoglycemic activity in rabbits with alloxan-induced diabetes. Cr­(III)-GCZ showed an early therapeutic response that persisted over an extended duration, while the Cr­(III)-GCZ-βCD inclusion complex showed a delayed therapeutic response but maintained an extended duration of activity compared with the reference (parent) drug, gliclazide (GCZ). The synthesized complexes exhibited good therapeutic potential, and the toxicity of these complexes was found to be quite low (high LD_50_ values).

## Graphical abstract

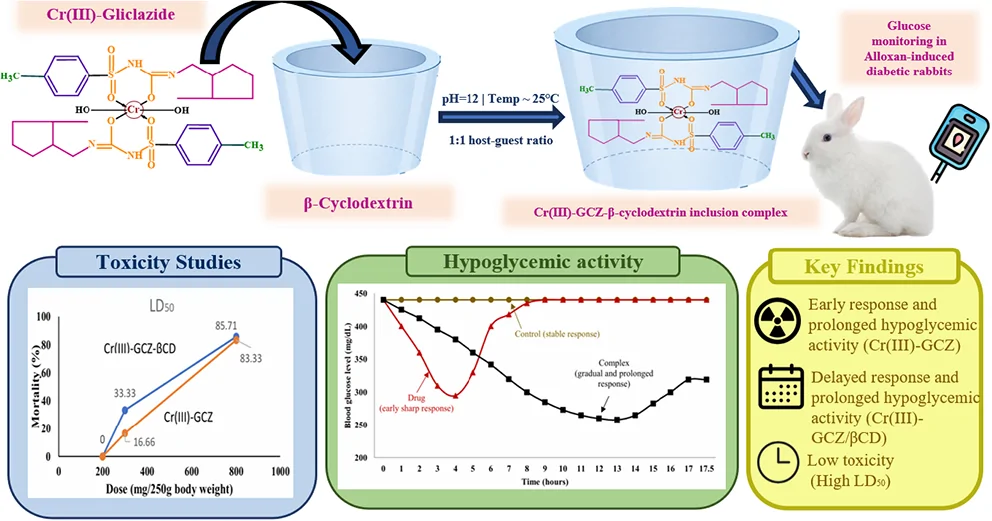

## 1 Introduction

Medicinal compounds containing heteroatoms, such as nitrogen, oxygen, and sulfur, have demonstrated potential as ligands when coordinated with iron metal centers. Upon complexation, the structural modification alters the pharmacological properties. Their therapeutic effects may be improved, diminished, or nullified. Metal chelates have exhibited pronounced antimicrobial activity in biological fluids and tissues and have the potential to target a broad spectrum of microorganisms and perform better than conventional antibiotics. Transition metal complexes and their derived drugs are promising candidates in the treatment of various diseases and are being tested in clinical trials.^1–4^

A limited number of chromium complexes with oral hypoglycemic drugs had been synthesized and evaluated for their biological (antibacterial and antidiabetic) activity, including the oat β-glucan-Cr­(III) complex and dipicolinic acid-metformin-Cr­(III).^5,6^ Very few studies have addressed the pharmacokinetic limitations, such as burst release and short-term action, that constrain their clinical utility.^7,8^ The pharmacological profile of existing antimicrobial drugs can be modified based on the well-established role of metal ions in the pathophysiology of diabetes and other diseases. Chromium, in its naturally occurring form as a dinicotinic acid-glutathione complex, commonly referred to as the glucose tolerance factor (GTF) is a key contributor to carbohydrate metabolism and enhances the effectiveness of insulin. Accordingly, it contributes to the regulation of blood sugar by preventing the excessive elevations in individuals susceptible to diabetic hyperglycemia as well as episodes of hypoglycemia. GTF chromium is also promoted as a nutritional supplement.^9^

Chromium’s involvement in nucleic acid metabolism and synthesis is suggested by its relatively high concentration in nuclear proteins compared to other transition metals and its deficiency depresses nucleic acid synthesis.^10^ This is also linked to increased levels of serum cholesterol and triglycerides, the development of aortic plaques, peripheral neuropathy, and corneal opacity. Moreover, insufficient chromium intake during pregnancy is associated with premature birth and intrauterine growth restriction, while in males, it has been connected to infertility.^11^ Consuming foods that naturally contain the GTF is considered the most effective way to obtain biologically active chromium, which plays a beneficial role compared to nutritional supplements.^12^ The GTF is found abundantly in brewer’s yeast and, to a lesser extent, in foods such as beef, chicken, lobster, etc.

Given chromium’s efficient role in insulin sensitization and metabolism of glucose, the combination of Cr­(III)-GCZ with a β-cyclodextrin (βCD) host offers a potential synergistic hypoglycemic activity from chromium that combines with sustained, controlled drug release afforded by host–guest inclusion chemistry.^13,14^ This research is centered on the synthesis, characterization, and evaluation of chromium metal complexes with gliclazide, a second-generation oral hypoglycemic sulfonylurea (SU) drug, further enhanced through a β-cyclodextrin inclusion complex designed to achieve prolonged hypoglycemic action. In addition to assessing antidiabetic potential, the study investigated the toxicity profile and evaluated the antibacterial properties of the prepared complexes. Rabbits were chosen as the experimental model due to their suitability for intravenous injection, oral administration via a feeding tube, and the ease of repeated blood sampling from the peripheral ear vein, all of which could be performed within a short time frame with minimal stress to the animals.

## 2 Materials and Methods

### 2.1 Synthesis of the Chromium­(III)-Gliclazide Complex

The GCZ ligand (2.23 g, 10 mmol) was combined with potassium hydroxide (0.56 g, 10 mmol) in a 1:1 ethanol–acetone mixture (100 mL) and heated under reflux with continuous stirring for 15 min. To this refluxing mixture, a solution of chromium­(III) acetate (3.76 g, 10 mmol), predissolved in a minimal volume of hot water, was added dropwise. Reflux was continued for a further 6 h, over which a green microcrystalline solid separated from the mixture. The solution was then concentrated to approximately 30 mL by slow evaporation on a water bath and cooled to room temperature. The precipitated complex was collected by filtration, washed successively with ethanol, acetone, and diethyl ether, and dried at 80 °C.

### 2.2 Synthesis of the β-Cyclodextrin Inclusion Complex of Chromium­(III)-Gliclazide

The Cr­(III)-GCZ complex (2.5 g, 4 mmol), dissolved in 5% NaOH, was added dropwise to an aqueous solution of βCD (4.44 g, 10 mmol) in 50 mL of water under constant stirring at room temperature. Stirring was continued for several hours, during which the initially greyish-green suspension darkened to a deep green color. The mixture was concentrated to about 20 mL by slow evaporation on a water bath at 60 °C, giving a dark-green microcrystalline product. The solid was isolated by filtration, washed with chloroform and acetone, and dried under vacuum at room temperature.

### 2.3 *In Vivo* Evaluation of Hypoglycemic Activity

Four groups of animals were used to assess the hypoglycemic activity of the Cr­(III)-GCZ-βCD inclusion compound. Group I served as the control and received 2 mL of distilled water (freshly prepared for each dose). Group II was given standard gliclazide (2 mg kg^–1^ body weight) as an aqueous solution/suspension. Groups III and IV were administered with the Cr­(III)-GCZ and Cr­(III)-GCZ-βCD compound, respectively.

### 2.4 Estimation of Blood Glucose Levels

Blood glucose concentration in each animal was monitored hourly from the time of dosing (0 h) up to 8 h postadministration for the control and standard drug groups, while monitoring was extended to 17 h for animals treated with the Cr­(III)-GCZ-βCD complex. Blood samples were withdrawn from the peripheral or midear vein of each animal, and glucose levels were determined using a one-touch glucometer.

### 2.5 Antibacterial Activity: Minimum Inhibitory Concentration (MIC)

The synthesized complexes were screened for antibacterial activity and expressed as the minimum inhibitory concentration (MIC).

#### 2.5.1 Preparation of Agar Plates

Stock solutions of the complexes were prepared in a minimal volume of dimethyl sulfoxide (DMSO) and diluted to 5 mL, followed by serial dilution in water/DMSO to give test concentrations of 10–1280 μg mL^–1^. Nutrient agar was prepared using these solutions, autoclaved, and poured (20 mL) into duplicate, prewarmed (40 °C) Petri dishes, which were labeled and refrigerated until use.

#### 2.5.2 Preparation of the Inoculum

Gram-positive and Gram-negative test organisms were subcultured on fresh nutrient agar and incubated at 37 °C for 24 h to obtain pure cultures and then grown in Mueller–Hinton broth at 37 °C for 24 h. Suspensions were standardized to 10^5^–10^6^ CFU cm^–3^ against the Kirby–Bauer standard and applied via a multipoint inoculator.

##### 2.5.2.1 Gram-Positive

*Escherichia coli* *(C)*, *Staphylococcus aureus*, coagulase-positive *Staphylococcus*, *E. coli*, and *Streptococcus*.

##### 2.5.2.2 Gram-Negative

*Pseudomonas (C)*, *E. coli*, *Salmonella typhi* *(R)*, *Pseudomonas*, *E. coli*, *Enterobacter cloacae*, *Enterococcus faecalis*, *Proteus mirabilis*, *Klebsiella pneumoniae*, and a sensitive *Salmonella* strain.

#### 2.5.3 Application of the Inoculum

Using a multipoint inoculator, batches of strains were applied to predried Mueller–Hinton agar plates containing the test compounds. Quality-control organisms were included as batch controls, and plain agar plates were inoculated before and after each compound batch to check for contamination and confirm inoculum viability. Plates were incubated at 37 °C for 18–24 h, and MIC values for the Gram-positive and Gram-negative organisms were recorded.

### 2.6 Toxicity (LD_50_) Studies

LD_50_ is the dose at which lethality occurs in half of the treated population, and it was determined for the synthesized complexes in albino rats (200–250 g) by administering graded doses and recording the resulting percentage mortality.

#### 2.6.1 Preparation of Complex Solutions

Accurately weighed amounts of each complex were dissolved in an appropriate solvent to give solutions/suspensions of 10, 20, 40, 80, and 160 mg mL^–1^. Solutions were administered orally via a feeding tube fitted with an automatic drencher. In the initial experiment, 30 rats were divided into 6 groups. The control group received 5 mL of solvent alone, while the five treatment groups received 5 mL of 10, 20, 40, 80, or 160 mg mL^–1^ solutions. Mortality and survival were recorded for each group, and LD_50_ values were calculated from the cumulative data using the Reed–Muench method.

## 3 Results and Discussion

Diabetes is a syndrome of chronic hyperglycemia with disturbances in metabolism due to insulin deficiency or resistance. Type 2 diabetes treatment includes a combination of diet, exercise, insulin, and oral medications, such as sulfonylureas (SUs). Second-generation SUs exhibit greater activity and effectiveness at lower doses due to improved β-cell absorption^15–17^ and are widely used for diabetes treatment. Researchers have attempted to modify these drugs by incorporating metal ions into their structures.^18,19^

### 3.1 Formation of the Inclusion Complex

Cyclodextrins form solid inclusion complexes (host–guest complexes) with a variety of molecules.^20,21^ In these complexes, the guest molecule resides within the cyclodextrin cavity.


1 Cr(III)‐GCZ‐βCD⇄Cr(III)‐GCZ+βCD

The host–guest ratio between β-cyclodextrin and Cr­(III)-GCZ, determined using Job’s method via circular dichroism, was found to be 1:1 (Figure 1a). Inclusion efficiency increased with pH, reaching its maximum at pH 12.5, which was selected for complex synthesis (Figure 1b). Temperature also influenced stability, with peak ellipticity at 25 °C, while the complex began degrading between 80 and 100 °C (Figure 1c).

**Figure 1:**
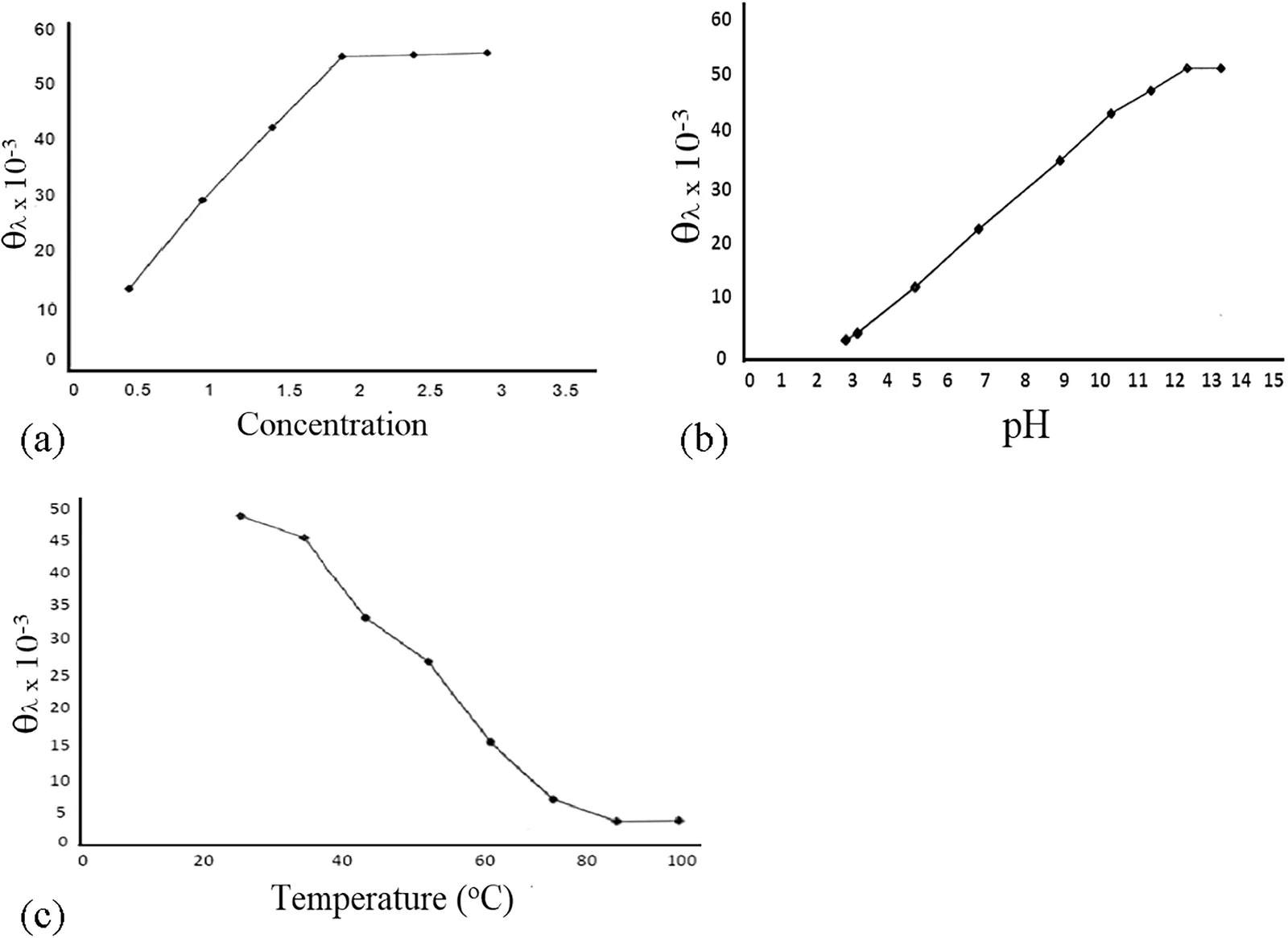
Effect of (a) concentration; (b) pH; and (c) temperature on the formation of the Cr­(III)-GCZ-βCD inclusion complex.

The elemental analysis (carbon, hydrogen, and nitrogen) for the Cr­(III)-GCZ complex, i.e., K_2_[Cr­(C_15_H_20_N_3_O_3_S)­(OAc)_2_(OH)_2_]·3H_2_O (Table 1), suggests an octahedral coordination geometry around the Cr­(III) ion, provided GCZ acts as a bidentate ligand (Figure 2). The inclusion complex fits in the following formula: K_2_[Cr­(C_15_H_20_N_3_O_3_S)­(OAc)_2_(OH)_2_]-[C_42_H_70_O_35_]·3H_2_O.

**Figure 2:**
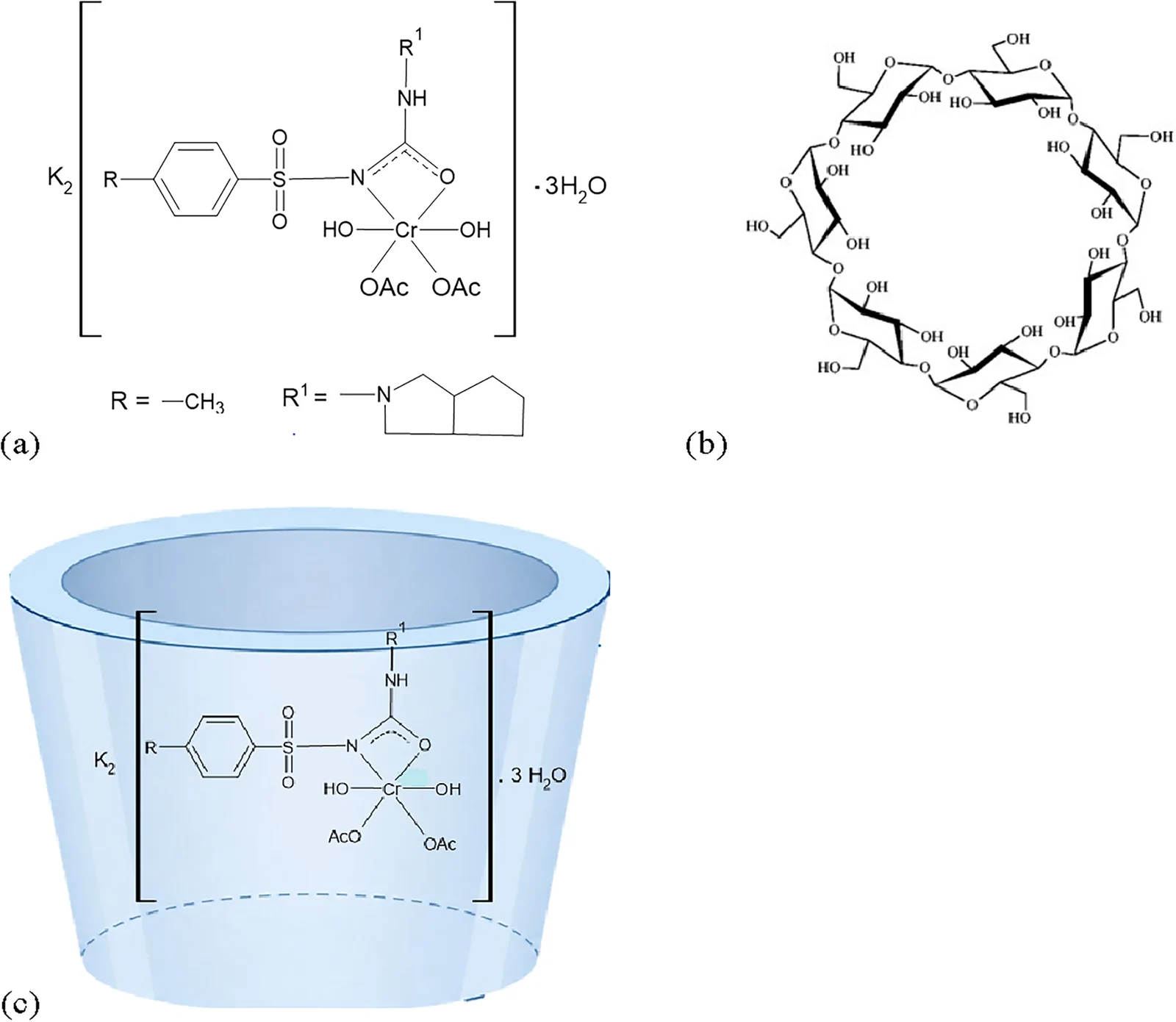
Chemical structure of (a) Cr­(III)-GCZ; (b) βCD; and (c) Cr­(III)-GCZ-βCD inclusion complex.

**Table 1:** Elemental Analysis Data

				elemental analysis (%)				
Sr. no	complex	color/physical state	Dec. Pt. (°C)	C	H	M	N	μ_eff_
1	K_2_[Cr(C_15_H_20_N_3_O_3_S)(OAc)_2_(OH)_2_]·3H_2_O	greyish green/amorphous	245–250	34.61	4.26	6.33	8.34	4.01
2	K_2_[Cr(C_15_H_20_N_3_O_3_S)(OAc)_2_(OH)_2_]-[C_42_H_70_O_35_]·23H_2_O	dark green/crystalline	255–260	32.36	4.67	2.21	2.83	

### 3.2 Circular Dichroism (CD) Spectral Studies of the Complex

The Cr­(III)-GCZ complex is an optically inactive compound and does not give any CD band. βCD is an optically active compound, but being colorless, it does not give any CD band in the visible region. However, a solution containing equimolar quantities of Cr­(III)-GCZ and βCD under appropriate experimental conditions gives a CD (circular dichroism) band in the visible region. Positive Cotton bands at 24,691 cm^–1^ (Δε = 20.103 degree L mol^–1^ cm^–1^) and 16,666 cm^–1^ (*A*ε = 26.6 × 10^–3^ degree L mol^–1^ cm^–1^) and a strong negative Cotton band at 19,230 cm^–1^ (*A*ε = 38.1 × 10^–3^ degree L mol^–1^ cm^–1^) were observed, Figure 3. The solution of the isolated solid Cr­(III)-GCZ-βCD complex in DMSO/water gave CD bands at the same position. The presence of a strong circular dichroism (CD) band in the visible region confirmed the successful formation of the Cr­(III)-GCZ-βCD inclusion complex.

**Figure 3:**
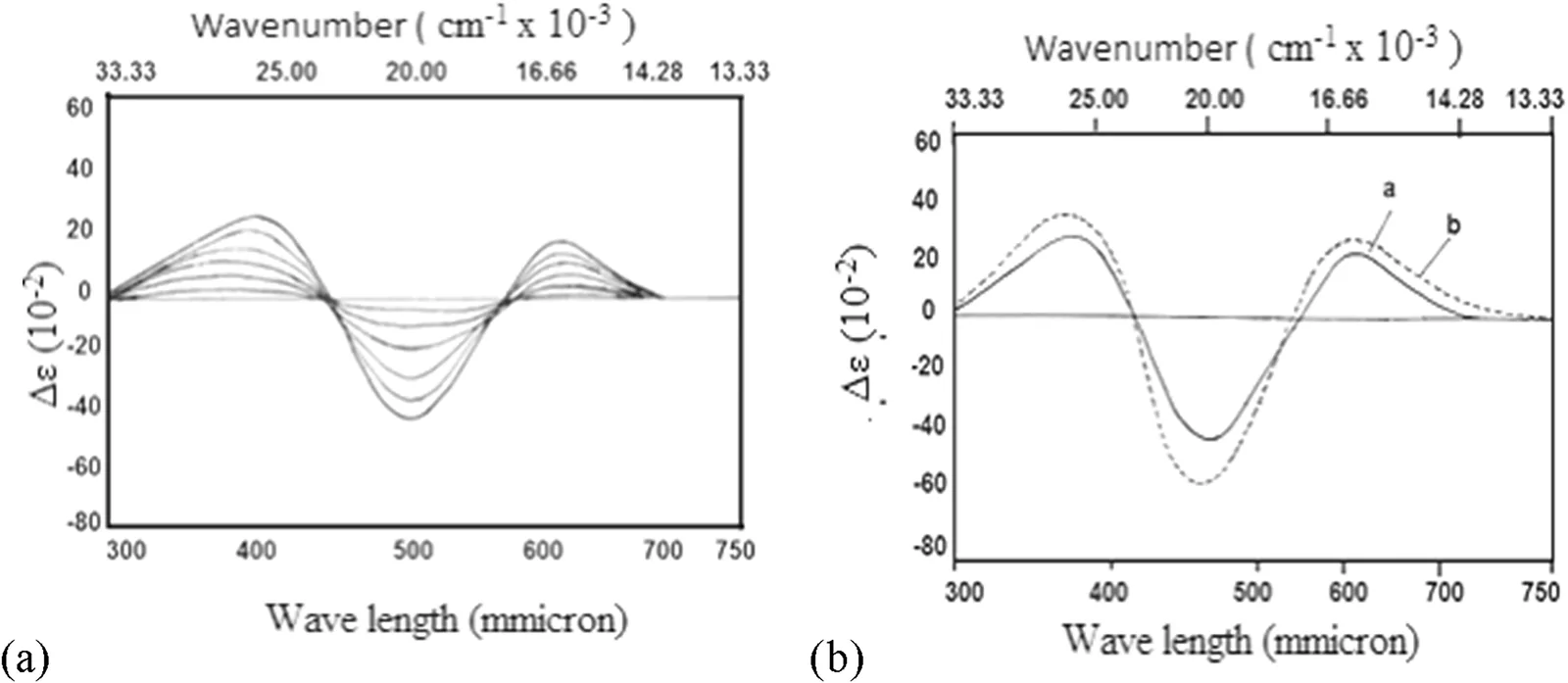
CD spectra of (a) isolated Cr­(III)-GCZ-βCD at different concentrations and CD spectra and (b) Cr­(III)-GCZ-βCD inclusion complex in solution.

### 3.3 Infrared Spectrum of the Synthesized Complexes

The IR spectrum of the Cr­(III)-GCZ-βCD complex shows a shift in the N–H stretching band from 3315 cm^–1^ (in Cr­(III)-GCZ) to 3325 cm^–1^ (Figure 4a,b). A broad O–H/water band appears at 3420 cm^–1^ in β-cyclodextrin, 3445 cm^–1^ in Cr­(III)-GCZ, and 3442 cm^–1^ in the inclusion complex. The C=O band shifts from 1640 cm^–1^ in Cr­(III)-GCZ to 1637 cm^–1^, a frequency also seen in β-cyclodextrin due to C–O linkage. These overlapping bands validate the synthesis of the inclusion complex.

**Figure 4:**
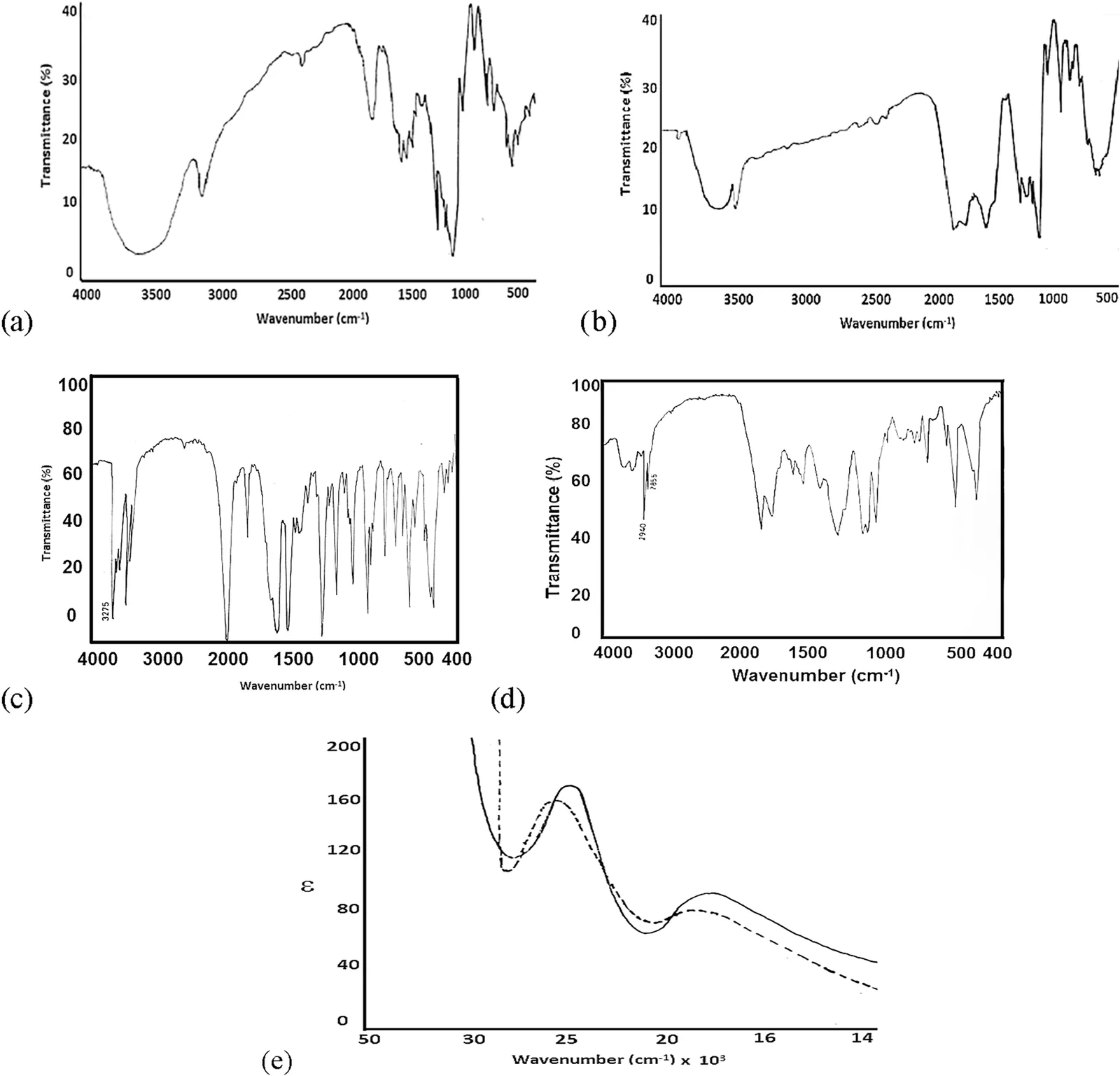
Solid-state FT-IR of (a) β-cyclodextrin; (b) Cr­(III)-GCZ-βCD; (c) gliclazide; (d) Cr­(III)-gliclazide complex; and (e) absorption spectra of the Cr­(III)-GCZ-βCD complex (solid line) and Cr­(III)-gliclazide (dotted line).

Comparison of the IR spectrum of the Cr­(III)-Gliclazide complex with that of the free ligand reveals noticeable changes, indicating coordination. The characteristic N–H stretching band at 3210 cm^–1^, along with the C=O (1704 cm^–1^) and C–N (1591 cm^–1^) bands of the free ligand, either shifts or disappears upon complex formation. In the IR spectrum of the Cr­(III)-Gliclazide complex, the N–H stretching band of the −SO_2_NHCO– group disappears, suggesting that the proton is replaced by the metal ion during coordination (Figure 4c,d). The C–N stretching band of the −SO_2_NCO– moiety shifts slightly from 1591 to 1593 cm^–1^. A broad, moderately strong band observed at 3445 cm^–1^ is of O–H stretching vibrations of attached water molecules. Additionally, the carbonyl C=O stretch shifts from 1704 cm^–1^ in the free ligand to 1640 cm^–1^ in the complex, further supporting coordination. This marked shift of C=O stretching indicates a bidentate mode of ligand coordination via the nitrogen atom and C=O group of the −SO_2_NCO– moiety (II).^22^

### 3.4 Coordination Modes of the Sulfonylurea Drugs (GCZ)

To locate the exact coordination sites and mode of coordination of these ligands in their complexes, they are characterized by various analytical techniques, i.e., infrared, ^1^H NMR, ^13^C NMR, absorption spectroscopy, etc.

#### 3.4.1 Absorption Spectrum of Synthesized Complexes

The absorption spectrum of the Cr­(III)-GCZ complex in DMSO (Figure 4e) contains two bands centered at 19,646 cm^–1^ (ε = 70 L mol^–1^ cm^–1^) and 25,641 cm^–1^ (ε = 150 L mol^–1^ cm^–1^) in the visible part of the spectrum. In addition to these bands, two very intense charge transfer bands are observed at 35,871 cm^–1^ (ε = 3970 L mol^–1^ cm^–1^) and 37,203 (ε = 2680 L mol^–1^ cm^–1^). Chromium­(III) is a d3 system with an F ground term. Cr­(III) complexes in an octahedral environment show three spin-allowed d–d bands at 17,400, 24,700, and 37,000 cm^–1^.^23^ The bands ν_1_, ν_2_, and ν_3_ are assigned to the transitions ^4^A_2g_ → ^4^T_2g_(F), ^4^A_2g_ → ^4^T_1g_(F), and ^4^A_2g_ → ^4^T_1g_(P), respectively.

All three spin-allowed transition bands may be observed when the ligand is transparent in the ultraviolet region of the spectrum. The intensity of ν_3_ is generally less than that of either of the other two spin-allowed bands, which agrees with its formation as a two-electron transition in a strong-field limit. In the present case, the observed band at 19,646 cm^–1^ may be assigned to the ^4^A_2g_ → ^4^T_2g_(F) transition, whereas the band at 25,641 cm^–1^ is attributed to the ^4^A_2g_ → ^4^T_1g_(F) transition. The third band may be obscured in the presence of a high-intensity charge-transfer band at 35,871 cm^–1^ extending from the UV region. Based on these observations, it is assumed that Cr­(III)-GCZ is a low-symmetry octahedral complex. It is further supported by the fact that absorption bands at 19,646 and 25,641 cm^–1^ are broad, a common feature of low-symmetry octahedral chromium­(III) complexes. A low molar absorptivity value (ε = 70–150) also indicates the octahedral geometry for the Cr-GCZ complex.^24^

The absorption spectrum of the inclusion complex in DMSO/H_2_O contains two bands in the visible region centered at 19,880 cm^–1^ (ε = 110 L mol^–1^ cm^–1^) and 24,953 cm^–1^ (ε = 140 L mol^–1^ cm^–1^). In addition to these bands, another high-energy band was also observed at 39,215 cm^–1^ (ε = 1150 L mol^–1^ cm^–1^). This band is due to βCD present in the inclusion complex, as βCD gives these high-energy bands in the UV region at 39,062 cm^–1^ (ε = 2124 L mol^–1^ cm^–1^) and 41,152 cm^–1^ (ε = 2110 L mol^–1^ cm^–1^). These observations show that d–d bands in Cr­(III)­GCZ complex are significantly shifted on the formation of the inclusion complex with βCD.

The magnetic moment study of such complexes further confirmed the octahedral structure. Usually, for octahedral Cr­(III) complexes, the spin-only value is very close to 3.88 μ_B_.^25,25^ The observed magnetic moment for the Cr­(III)-GCZ complex is 4.01 μ_B_, and this indicates three unpaired electrons in this complex and confirms an octahedral structure for the Cr­(III)-GCZ complex.

### 3.5 ^1^H NMR Spectroscopy

The ^1^H NMR spectrum of gliclazide in DMSO-*d* (Figure 5a) is relatively easy to interpret due to its simple structure. The most upfield signal was assigned to heterocyclic ring protons. This signal appears as a multiplet around 1.2–1.7 ppm due to the coupling of several protons of the heterocyclic ring. The complex splitting pattern made it difficult to make definite assignments of these signals. The N–H proton has no neighboring proton and is deshielded more. As such, it gave a broad signal at 8.1 ppm. The NO–H proton of the −SO_2_NHCO– moiety is the most deshielded. The signal due to this proton shifted to the downfield region at 10.0 ppm. The distinct peaks at 7.6 ppm (a doublet) and 7.4 ppm (a doublet) are due to aromatic protons H_1_, H_2_, H_3_, and H_4_. The most upfield signal (a singlet) at 2.4 ppm is assigned to the CH_3_ group.^26^

**Figure 5:**
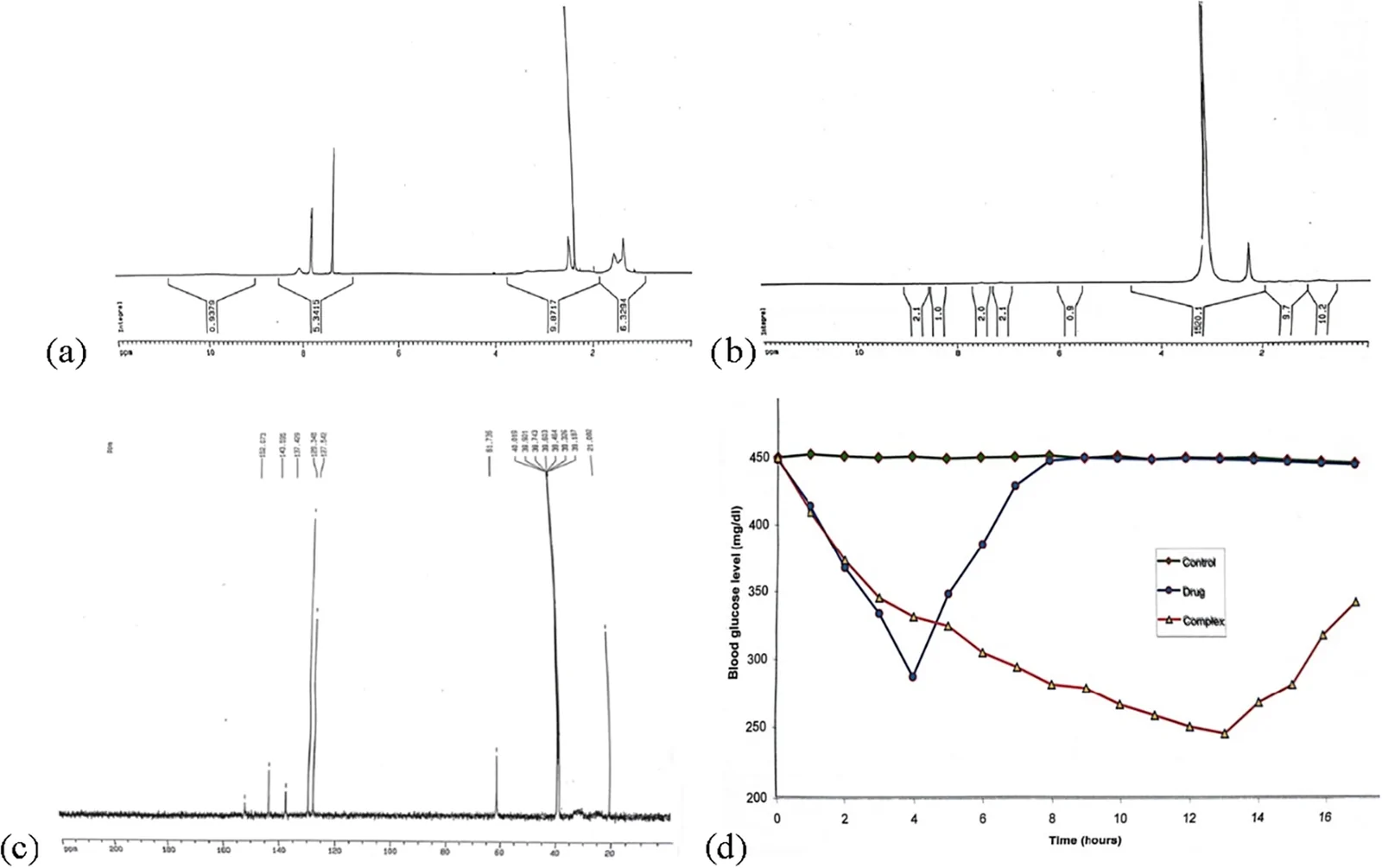
^1^H NMR spectrum of (a) gliclazide; (b) Cr­(III)-GCZ complex in DMSO-*d*_6_; (c) ^13^C NMR spectrum of the gliclazide (GCZ) ligand; and (d) change in the mean blood level (mg dL^–1^) of alloxan diabetic rabbits treated with the gliclazide (GCZ) drug and Cr­(III)-GCZ-βCD complex.

The major characteristic of the ^1^H NMR spectra of all these ligands is the position of the N–H proton of the −SO_2_NHCO– group. This proton is directly linked with electronegative nitrogen; its signal is shifted to the most downfield, at around 10.0 ppm. When a metal ion coordinates through this nitrogen, the signal due to the N–H proton disappears. This observation supports the prediction based on IR spectra that the nitrogen atom of the −SO_2_NHCO– moiety is involved in coordination with metals. However, the presence of some ligand peaks, especially in the ^1^H NMR spectrum of Cr­(III)-GCZ complexes (Figure 5b), indicates the formation of this complex. The signal N^b^–H proton has disappeared in the Cr­(III)-GCZ complex, indicating the involvement of the SO_2_N^b^CO– group in coordination.

### 3.6 ^13^C NMR Spectroscopy

The ^13^C NMR chemical shifts of various resonances were assigned based on the electronegativity relationship and by running the distortionless enhancement by polarization transfer (DEPT) spectrum using the 135° pulse sequence.

The upfield or, in some cases, the downfield shift of these signals in the spectra of complexes of these ligands shows that the carbonyl group of the −SO_2_NHCO– moiety in the ligand is associated in coordination with metals. This finding provides additional evidence for the prediction based on IR spectra that the carbonyl group (C=O) is involved in coordination. It validates the monodentate and bidentate coordination modes of SUS ligands (I–V). Clear NMR spectra of Cr­(III)-GCZ complexes could not be obtained because of their low solubility and paramagnetic nature.

The ^13^C NMR spectrum of gliclazide in DMSO-*d* (Figure 5c) shows the most downfield resonance due to carbonyl carbon C at 152.07 ppm. It is shifted upfield or downfield in coordination with metal through the carbonyl group (Figure 5c). The resonance line of aromatic carbons appears between 127.54 and 143.59 ppm. Among the four aromatic ring signals, the two with low intensity are due to quaternary carbons because of their large relaxation times. These carbons gave signals at 143.59 and 137.42 ppm, respectively. The cyclohexyl ring carbon (C_2_–C_3_) signal appears as broad peaks from 20 to 40 ppm. The carbon atom in the cyclohexyl ring is directly attached to the more electronegative nitrogen atom. The signal due to this carbon atom is shifted downfield to 61.63 ppm.

### 3.7 Pharmacological Studies

#### 3.7.1 Hypoglycemic Activity

The hypoglycemic activity of Cr­(III)-GCZ and Cr­(III)-GCZ-βCD was evaluated in comparison with the standard drug (GCZ) and control groups of alloxan-induced diabetic rabbits. A dose of 1 mg kg^–1^ body weight was found to be sufficient to produce hypoglycemia in animals. The changes in the mean blood glucose level (BGL) of control and treated groups of animals are reported (Table 2) (Figure 5d).

**Table 2:** Change in the Mean Blood Glucose Level (mg dL^–1^) of Alloxan Diabetic Rabbits Treated with (1 mg kg^–1^ b.w.) the Gliclazide Drug and Cr­(III)-GCZ-β-Cyclodextrin Inclusion Complex

time after administration (h)	alloxan diabetic control	alloxan diabetic rabbits treated with standard GCZ	alloxan diabetic rabbits treated with Cr(III)-GCZ-βCD	alloxan diabetic rabbits treated with Cr(III)-GCZ
0	449 ± 2.00	447.20 ± 6.14	448.40 ± 4.09	450 ± 3.80
1	451 ± 2.00	412.60 ± 3.20	408.00 ± 2.91	417 ± 3.24^c^
2	449 ± 4.5	366.20 ± 7.33	371.80 ± 5.22	363 ± 3.05
3	447.8 ± 2.0	332.2 ± 4.90	343.80 ± 2.671^c^	317 ± 4.02^a^
4	448.6 ± 5.44	285.80 ± 3.81	329.80 ± 1.87^a^	270 ± 3.97^a^
5	447.2 ± 5.89	346.80 ± 5.03	323.20 ± 3.28^b^	226 ± 2.16^a^
6	448.2 ± 1.14	383.80 ± 3.04	303.80 ± 2.86^a^	284 ± 2.25^a^
7	448.8 ± 4.03	427.4 ± 7.50	293.40 ± 1.81^a^	354 ± 5.43^a^
8	449.9 ± 6.12	446.00 ± 9.98	280.60 ± 3.48^a^	402 ± 3.87^a^
9	448.00 ± 6.12	448.20 ± 8.05	277.8 ± 4.08^a^	
10	449.60 ± 5.57	447.60 ± 7.51	265.80 ± 3.68^a^	
11	447.00 ± 4.18	446.80 ± 7.41	258.00 ± 4.52^a^	
12	448.40 ± 5.44	447.60 ± 6.51	250.00 ± 4.52^a^	
13	448.00 ± 5.24	447.00 ± 6.70	245.2 ± 4.28^a^	
14	448.60 ± 6.17	446.4 ± 7.36	267.8 ± 3.88^a^	
15	447.00 ± 5.78	445.6 ± 6.64	281.00 ± 12.18^a^	
16	446.20 ± 5.85	444.8 ± 6.51	317.60 ± 3.04^a^	
17	445.20 ± 5.64	444.00 ± 6.81	342.2 ± 5.60^a^	

a *p* < 0.001 significant with respect to GPZ.

b *p* < 0.01 significant with respect to GPZ.

c *p* < 0.05 significant with respect to GPZ.

On administering Cr­(III)-GCZ and Cr­(III)-GCZ-βCD to rabbits with alloxan diabetes, a major fall in the mean BGL was seen. Up to 2 h, the decrease in the BGL of both the complexes and drug (GCZ)-treated groups was nearly the same. After 4 h, the BGL of Cr­(III)-GCZ, Cr­(III)-GCZ-βCD, and drug-loaded groups was 270.60 ± 3.97, 329.80 ± 1.87, and 285.60 ± 3.57, respectively, compared to the control (448.6 ± 5.44), indicating a decrease in the BGL of the drug more than in the Cr­(III)-GCZ-βCD-treated group. At this stage, the parent drug becomes deactivated, and BGL begins to rise steeply and reaches its maximum at 8 h. However, the complex (Cr­(III)-GCZ) remained active and showed a significant decrease in BGL for up to 5 h, after which it began to rise. At 5 h, the decrease in BGL was much greater in the Cr­(III)-GCZ complex (226.00 ± 3.16) than in GCZ (346.80 + 5.03). After 6 h, the BGL of the Cr­(III)-GCZ group (284.00 ± 2.25) and GCZ-loaded group (383.80 ± 3.04) increased. A significant difference in antidiabetic activity between the two continued until 8 h, when the BGL of the GCZ drug-loaded group (446.00 ± 9.98) became almost equal to the control group (449.9 ± 6.12), whereas the Cr­(III)-GCZ complex was still lowering the glucose level (402.00 ± 3.87).

Both compounds, GCZ drug and Cr­(III)-GCZ complex, showed hypoglycemic activity under similar experimental conditions. However, at the end of 4 h, the decrease in the glucose level was comparatively greater in the case of the Cr­(III)-GCZ complex (∼36%) than the drug (∼40%). After 4 h, the drug becomes inactive, while the Cr­(III)-GCZ complex continues lowering the glucose level. At the end of the fifth hour, the difference in the BGL further increases (GCZ drug: ∼22% and Cr­(III)-GCZ complex: ∼50%), after which the Cr­(III)-GCZ complex also becomes inactive.

In contrast, after 6 h, the BGL of the inclusion complex (Cr­(III)-GCZ-βCD) group (303.80 ± 2.86) was less than the drug-treated group (383.80 ± 3.04). After 8 h, its BGL was 280.60 ± 3.48, and that of the drug group was 446.00 ± 9.98 (close to the control group: 449.9 ± 6.12). At this stage, the drug has lost its action, but the complex still lowers the glucose level. The lowering of the BGL continued, and after 13 h, the difference between the BGL of the control (448.00 ± 5.24) and Cr­(III)-GCZ-βCD-treated group (245.2 ± 4.28) was quite significant. Even after 17 h, the inclusion complex still exhibited a hypoglycemic effect compared with the control group (445.2 ± 5.64) and the drug-treated group (342 ± 2.56). These observations revealed that the inclusion complex has great potential to lower the BGL and remains effective for a longer time. After 4 h, the fall in the glucose level was greater in the case of the drug (36%) than the inclusion complex (26%). However, after 6 h, the decrease in the blood glucose level in the complex-treated group was greater (32%) than in the drug-treated group (15%). The drug becomes completely inactive after 8 h of its administration, but the inclusion complex remains active and continues lowering the BGL until 13 h. At this stage, the decrease in the glucose level of the inclusion complex-treated group was quite significant (45%). It remains active in keeping the BGL at a much lower level compared to the parent drug even after 17 h (Figure 5d).

The hypoglycemic potential and duration of action of the Cr­(III)-GCZ-βCD inclusion complex are greater than those of GCZ and the Cr­(III)-GCZ complex. This may be attributed to the formation of a distinct Cr­(III)-GCZ-βCD compound where the weak binding of Cr­(III)-GCZ to βCD within the inclusion complex enables slow and controlled release *in vivo*, resulting in enhanced and prolonged hypoglycemic activity. Moreover, seven glucopyranose units linked in a ring configuration and three similar units in the acarbose molecule (an antidiabetic drug), with a difference in the CH_2_OH group in the βCD molecule, may benefit the inclusion compound, with such significant activity than Cr­(III)-GCZ.

The hypoglycemic effect of this compound is either a consequence of increased potential for insulin secretion or probably due to an increase in the peripheral utilization of glucose or a decrease in glucose absorption in the gut.

### 3.8 Toxicity

#### 3.8.1 Toxicity (LD_50_) of Metal Complexes of Gliclazide

Most drugs employed in clinical therapy are associated with undesirable adverse effects; however, the selective toxicity exhibited by certain chemical and biological substances enables their use as effective therapeutic agents.^27^ Studies related to toxicity usually expressed in terms of LD_50_ were performed to find their therapeutic index. Reed and Muench devised a simple method for estimating the toxicity level of chemical or biological substances, which has also been employed for certain diagnostic assays. It has been reported that toxic activities for compounds are considered significant when LD_50_ is >30 μg mL^–1^.^28^

The LD_50_ value for the Cr­(III)-GCZ complex was 2.26 g kg^–1^ b.w., orally in rats. The LD_50_ value for the ligand (GCZ) is observed to be greater than 3 g kg^–1^ b.w. The complex, however, appears to be more toxic than the drug. Besides this, a normal dose of 1 mg kg^–1^ b.w., orally in 25 rats, showed no signs of toxicity. This observation suggested that Cr­(III)-GCZ was not toxic at the drug’s normal dose (1 mg kg^–1^ b.w.). However, the LD_50_ value for Cr­(III)-GCZ-βCD was found to be 2.65 g kg^–1^ b.w. in rats (Table 3). This indicates that the inclusion complex is about 1.13 times less toxic than the Cr­(III)-GCZ complex and was found to be less toxic than most of the complexes synthesized in this work.

**Table 3:** Determination of Toxicity LD_50_ of Cr­(III)-βCD (I) and Cr­(III)-GCZ (II) by the Reed–Muench Method

			No. of animals after 7 days				mortality (%)		LD_50_ ^a^ (g kg^–1^)	
			Cr(III)-GCZ-βCD (I)		Cr(III)-GCZ (II)					
group	dose (mg)/250 g b.w.	No. of animals	dead	survived	dead	survived	(I)	(II)	(I)	(II)
1	800	5	4	1	4	1	85.71	83.33	2.56	2.26
2	400	5	2	3	1	4	33.33	16.66		
3	200	5	0	5	0	5	0.00	0.00		
4	100	5	0	5	0	5	0.00	0.00		
5	50	5	0	5	0	5	0.00	0.00		

a 95% confidence interval.

### 3.9 Antibacterial Activity of the Metal Complexes

Cr­(III)-GCZ was screened for its antibacterial properties against few Gram-positive and Gram-negative bacteria. The antibacterial studies were performed in DMSO water solution in the concentration range of 10–1280 μg cm^–3^. The minimum inhibitory concentration (MIC) data of Gram-negative bacteria are in Table 4. No significant effect was observed against selected Gram-positive bacteria, and a negative zone of inhibition of organisms was obtained against standard streptomycin in sulfate. The antibacterial activity of the complex was evaluated using one concentration of this complex, 100 μg cm^–3^ against different microorganisms, and then they were compared with standard antidiabetic streptomycin sulfate (Table 4).

**Table 4:** MIC Data of the Metal Complex against Gram-Negative Bacteria (μg cm^–3^)­^a^

compound	1	2	3	4	5	6	7	8	9
Cr-GCZ (Gram-negative bacteria)	1280	1280	1280	1280			1280		

a 1 = Pseudomonas (C), 2 = *E. coli* (C), 3 = *S. typhi* (R), 4 = *E. coli*, 5 = *E. cloacae*, 6 = *E. faecalis*, 7 = *P. mirabilis*, 8 = *K. pneumoniae*, and 9 = *Salmonella-*sensitive.

## 4 Conclusions

The pharmacological profile of Gliclazide was modified based on the well-established role of chromium ions in the pathophysiology of diabetes. A transition metal complex was synthesized with the currently available second-generation oral hypoglycemic sulfonylurea drug (gliclazide). An inclusion compound of the Cr-gliclazide complex with β-cyclodextrin has also been synthesized to enhance the hypoglycemic activity of selected drug and displayed delayed therapeutic response while maintaining an extended duration of activity and exhibited good therapeutic potential with low toxicity.

## References

[1] Makvandi P., Wang C. Y., Zare E. N., Borzacchiello A., Niu L. N., Tay F. R. (2020). Metal-based nanomaterials in biomedical
applications: Antimicrobial activity and cytotoxicity aspects. Adv. Funct. Mater..

[2] Millanao A. R., Mora A. Y., Villagra N. A., Bucarey S. A., Hidalgo A. A. (2021). Biological
effects of quinolones: A family of broad-spectrum antimicrobial agents. Molecules.

[3] Yousuf I., Bashir M., Arjmand F., Tabassum S. (2021). Advancement
of metal
compounds as therapeutic and diagnostic metallodrugs: Current frontiers
and future perspectives. Coord. Chem. Rev..

[4] Giorgi E., Binacchi F., Marotta C., Cirri D., Gabbiani C., Pratesi A. (2023). Highlights of new strategies
to increase the efficacy
of transition metal complexes for cancer treatments. Molecules.

[5] Li P., Wang Y., Wang X., Li R., Wang K., Jiang Y., Zhang M., Huang C., Ma Q., Sun J., Quan J. (2024). Preparation of a Novel Oat *β*-Glucan–Chromium­(III) Complex and Its Hypoglycemic Effect
and Mechanism. Molecules.

[6] Dong J. I., Wen B., Song Z., Chai J., Liu B., Tian W. J., Liang G., Yang B. (20121). Potential Antidiabetic Molecule Involving
a New Chromium­(III) Complex of Dipicolinic Acid and Metformin as a
Counter Ion: Synthesis, Structure, Spectroscopy, and Bioactivity in
Mice. Arabian J. Chem..

[7] Ragole V. D., Wankhede D. S., Gayakwad S. V. (2022). Biologically
active mixed ligand
complexes of Co­(II), Ni­(II), Cu­(II) and Zn­(II) as potential antimalarial,
antidiabetic and anticancer agents. Inorg. Nano-Met.
Chem..

[8] Javaid A., Akbar I., Javed H., Khan U., Iftikhar H., Zahra D., Rashid F., Ashfaq U. A. (2021). Role of
heavy metals
in diabetes: Mechanisms and treatment strategies. Crit. Rev. Eukaryotic Gene Expression.

[9] Pala R., Sari M. A., Erten F., ER B., Tuzcu M., Orhan C., Sahin K. (2020). The effects
of chromium
picolinate on glucose and lipid metabolism in running rats. J. Trace Elem. Med. Biol..

[10] Renu K., Chakraborty R., Myakala H., Koti R., Famurewa A. C., Madhyastha H., Gopalakrishnan A. V. (2021). Molecular mechanism
of heavy metals (Lead, Chromium, Arsenic, Mercury, Nickel and Cadmium)-induced
hepatotoxicity – A review. Chemosphere.

[11] Wróblewski M., Wróblewska W., Sobiesiak M. (2024). The role of selected elements in
oxidative stress protection: Key to healthy fertility and reproduction. Int. J. Mol. Sci..

[12] Yao X., Liu R., Li X., Li Y., Zhang Z., Huang S., Yang X. (2021). Zinc, selenium and chromium co-supplementation improves
insulin resistance by preventing hepatic endoplasmic reticulum stress
in diet-induced gestational diabetes rats. J.
Nutr. Biochem..

[13] Nicolaescu O. E., Belu I., Mocanu A. G., Manda V. C., Rău G., Pîrvu A. S., Ionescu C., Ciulu-Costinescu F., Popescu M., Ciocîlteu M. V. (2025). Cyclodextrins:
Enhancing Drug Delivery,
Solubility and Bioavailability for Modern Therapeutics. Pharmaceutics.

[14] Hoti G., Bajwa N., Caldera F., Singh P. A., Hussein I., Cecone C., Matencio A., Spagnolo R., Argenziano M., Cavalli R., Madan J., Trotta F. (2025). Cyclodextrin-based
therapeutics delivery systems: A review of current clinical trials. Curr. Res. Pharmacol. Drug Discovery.

[15] He Z. X., Zhou Z. W., Yang Y., Yang T., Pan S. Y., Qiu J. X., Zhou S. F. (2015). Overview
of clinically approved oral
antidiabetic agents for the treatment of type 2 diabetes mellitus. Clin. Exp. Pharmacol. Physiol..

[16] Al-Saleh Y., Sabico S., Al-Furqani A., Jayyousi A., Alromaihi D., Ba-Essa E., Alawadi F., Alkaabi J., Hassanein M., Al-Sifri S., Saleh S. (2021). Sulfonylureas in the
current practice of type 2 diabetes management: are they all the same?
Consensus from the Gulf Cooperation Council (GCC) countries advisory
board on sulfonylureas. Diabetes Ther..

[17] Alhadramy M. S. (2016). Diabetes
and oral therapies: A review of oral therapies for diabetes mellitus. J. Taibah Univ. Med. Sci..

[18] Levina A., Lay P. A. (2011). Metal-based anti-diabetic
drugs: Advances and challenges. Dalton Trans..

[19] El-Megharbel S. M., Al-Baqami N. M., Al-Thubaiti E. H., Qahl S. H., Albogami B., Hamza R. Z. (2022). Antidiabetic
drug sitagliptin with divalent transition
metals manganese and cobalt: Synthesis, structure, characterization,
antibacterial and antioxidative effects in liver tissues. Curr. Issues Mol. Biol..

[20] Mura P. (2015). Analytical
techniques for characterization of cyclodextrin complexes in the solid
state: A review. J. Pharm. Biomed. Anal..

[21] Ribeiro A., Figueiras A., Santos D., Veiga F. (2008). Preparation and solid-state
characterization of inclusion complexes formed between miconazole
and methyl-β-cyclodextrin. AAPS PharmSciTech.

[22] Raman N., Kulandaisamy A., Jeyasubramanian K. (2001). Synthesis, spectroscopic characterization,
redox, and biological screening studies of some Schiff base transition
metal (II) complexes derived from salicylidene-4-aminoantipyrine and
2-aminophenol/2-aminothiophenol. Synth. React.
Inorg. Met.-Org. Chem..

[23] Cotton, F. A. ; Wilkinson, G. ; Murillo, C. A. ; Bochmann, M. Advanced Inorganic Chemistry; John Wiley & Sons, 1999.

[24] Rodrigues J. A. O., Neto J. O., Santos C. C., Nogueira C. E. S., DE
Sousa F. F., DE Menezes A. S., Dos Santos A. O. (2022). Phase changes
of tris­(glycinato) chromium (III) monohydrate crystal systematically
studied by thermal analyses, XRPD, FTIR, and Raman combined with ab
initio calculations. Spectrochim. Acta, Part
A.

[25] Attia A. S. (2007). Synthesis
and spectroscopic studies on chromium (III) complex containing mixed-valence
chrysenesemiquinone-chrysenecatecholate ligands and 2,2′-bipyridine
coligand. Spectrochim. Acta, Part A.

[26] Moyano J. R., Arias-Blanco M. J., Gines J. M., Rabasco A. M., Pérez-Martínez J. I., Mor M., Giordano F. (1997). Nuclear magnetic resonance investigations of the inclusion
complexation of gliclazide with β-cyclodextrin. J. Pharm. Sci..

[27] Cope, W. G. ; Leidy, R. B. ; Hodgson, E. Classes of Toxicants: Use Classes. In A Textbook of Modern Toxicology; John Wiley and Sons Inc., 2004; pp 49–74.

[28] Reed L. J., Muench H. (1938). A Simple Method of Estimation Fifty
Percent End Points. Am. J. Epidemiol..

